# Optimizing the diagnosis of pelvic lymph node metastasis in bladder cancer using computed tomography and magnetic resonance imaging

**DOI:** 10.1186/s40880-018-0271-6

**Published:** 2018-03-12

**Authors:** Thomas B. L. Lam

**Affiliations:** Academic Urology Unit, Division of Applied Health Sciences, University of Aberdeen, Aberdeen, AB25 2ZD Scotland UK

Bladder cancer (BCa) is one of the most common malignant urogenital tumors in the world, and is especially common in China [1]. An important component of BCa staging is the determination of pelvic lymph node status, which provides valuable prognostic information and influences treatment decisions [2]. Cross-sectional imaging techniques such as computed tomography (CT) and magnetic resonance imaging (MRI) have proven to be useful for detecting malignant involvement of the pelvic lymph nodes. Both methods rely on morphological criteria, specifically, size and shape, as a predictor of lymph node metastasis [3]. Over the past decade, tremendous advances in CT and MRI technology, including the introduction of diffusion-weighted and ultra-small, superparamagnetic-particle, iron-oxide-enhanced MRI techniques, have greatly improved imaging resolution, thus readily revealing lymph nodes with diameters as small as 3.0 mm. In addition, the small intestine can be examined without inflation and small veins can be easily distinguished from lymph nodes [4, 5]. However, a clear consensus regarding the new criteria for imaging-based lymph node evaluation is lacking, and the two modalities must once again be assessed for their ability to detect metastatic lymph nodes. In a study recently published in the *Chinese Journal of Cancer*, titled “Computed tomography and magnetic resonance imaging evaluation of pelvic lymph node metastasis in bladder cancer,” Li et al. [6] analyzed the diagnostic accuracy of CT and MRI. Using pelvic lymph node dissection and histopathology as the reference standard, they were able to establish optimal diagnostic criteria.

In their study, the authors retrospectively examined the imaging characteristics of 191 BCa patients who underwent radical cystectomy. Data on the size, shape, density, and diffusion of the lymph nodes on CT and/or MRI were obtained and analyzed. Lymph node metastasis was pathologically diagnosed in 47/191 (24.6%) patients. Metastases were detected in 184 of the 3317 resected lymph nodes, mainly in those of the perivesicular, external iliac, internal iliac, and obturator regions. Among the imaging-detectable lymph nodes, 51/82 (62.2%) were confirmed to be positive for metastasis. The detection rate of metastatic nodes increased as the tumor stage increased. Lymph nodes with a short-axis diameter of < 3.0 mm were rarely seen on CT and/or MRI. The receiver operating characteristic (ROC) curve analysis showed that a short diameter of 6.8 mm was the optimal threshold for the diagnosis of metastatic lymph nodes, based on an area under the ROC curve of 0.815, a sensitivity of 83.0%, a specificity of 64.3%, and a Youden index of 47.3%. Imaging signs such as the fatty hilum of the lymph node and a short/long-axis diameter ratio ≤ 0.4 were usually characteristic of non-metastatic lymph nodes, while spiculated margins and necrosis were commonly observed in metastatic lymph nodes.

Size is a well-established and important index for detecting malignancy in the pelvic lymph nodes. In general, a smaller threshold values indicates a higher sensitivity and lower specificity; and a larger threshold a lower sensitivity and higher specificity. Although, by convention, a short-axis lymph-node diameter of 10 mm is considered the threshold value for malignancy on both CT and MRI [7, 8], several recent studies demonstrated that a cutoff value of 10 mm was not appropriate [9, 10]. The optimal cutoff value of 6.8 mm determined by Li et al. [6] has the potential to modify current diagnostic and treatment algorithms for BCa. In addition to lymph node size, several imaging characteristics were identified as indicative of benign changes in the lymph nodes, such as a short/long-axis lymph node diameter ratio of ≤ 0.4 and the presence of a fatty hilum. Other characteristics correlated with lymph node metastasis, such as spiculate or obscure margin and necrosis.

The limitations of the study were the relatively small number of patients enrolled (n = 191), the high attrition rate (< 80% of patients who underwent standard pelvic lymphadenectomy were enrolled), and the retrospective study design. Nevertheless, the findings will contribute to the discussion on the criteria for the diagnosis of metastatic lymph nodes in BCa and should encourage future prospective studies to validate them.
